# Oral Biofluid Biomarker Research: Current Status and Emerging Frontiers

**DOI:** 10.3390/diagnostics6040045

**Published:** 2016-12-17

**Authors:** Austin Wang, Chris P. Wang, Michael Tu, David T.W. Wong

**Affiliations:** School of Dentistry, University of California at Los Angeles, Los Angeles, CA 90095, USA; wang25austin@ucla.edu (A.W.); chriswang9409@gmail.com (C.P.W.); emtu@ucla.edu (M.T.)

**Keywords:** salivary diagnostics, noninvasive, biomarkers

## Abstract

Salivary diagnostics is a rapidly advancing field that offers clinicians and patients the potential of rapid, noninvasive diagnostics with excellent accuracy. In order for the complete realization of the potential of saliva, however, extensive profiling of constituents must be conducted and diagnostic biomarkers must be thoroughly validated. This article briefly overviews the process of conducting a study of salivary biomarkers in a patient cohort and highlights the studies that have been conducted on different classes of molecules in the saliva. Emerging frontiers in salivary diagnostics research that may significantly advance the field will also be highlighted.

## 1. Introduction

Molecular diagnostics is the collection of techniques used to analyze and monitor biological markers associated with diseases. In the field of molecular diagnostics, this involves intensive studies of patient cohorts in order to discover and validate the molecular constituents that make a diseased patient differ from a healthy individual. Salivary diagnostics is a rapidly advancing field in molecular diagnostics. Studies have shown that saliva contains various molecular compounds, such as nucleic acids and proteins that have been linked to abnormalities and complications from illnesses [1,2]. Currently, scientists have shown that these salivary constituents are effective indicators of many disorders. Multiple proteomic, transcriptomic, and microbiological markers have been identified for various pathologies: some prominent studies have been oral cancer, breast cancer, lung cancer, pancreatic cancer, and Sjögren’s syndrome [3,4,5,6].

In addition to the fact that research is readily demonstrating saliva as a viable biofluid for performing detection, it is also readily evident that saliva has many practical advantages compared to traditional diagnostic mediums of performing tests for disease diagnostics. In comparing the workflow for blood-based collection to saliva-based collection, Yoshizawa et al. [3] notes that there are some clinical advantages to using saliva:

1. Saliva collection is undemanding: procurement of saliva does not require highly trained personnel, and can be performed easily and readily, in contrast with blood sampling. To obtain sample saliva, expensive tools are not necessary.
2. Saliva collection is noninvasive: Individual patients are usually more comfortable with saliva sampling, and are more likely to participate.
3. Saliva samples are easier to handle and store: secretions in saliva that are not present in serum or plasma help decrease the risk of HIV transmission, and saliva does not clot.

Ultimately, the selection of saliva when compared to other diagnostic biofluids will be based on both the specific molecular constituents that are targeted and the practicalities of sample collection and processing. For example, urine is a molecularly rich biofluid that is useful in various diagnostic [7,8] scenarios and can be collected relatively easily. However, urine may be inferior for microRNA-based disease detection, as saliva has been found to have a lower microRNA content than urine or cerebrospinal fluid [9]. Healthcare professionals and scientists must work in cooperation to holistically determine the most compelling biofluid for performing diagnostic tests.

This review presents the current state of saliva diagnostics, broadly overviewing investigations into salivary constituents and considerations that must be made in the process. We will finally discuss the validation of obtained biomarkers by consulting the six existing “omics” libraries: proteome, transcriptome, immunome, microbiome, metabolome, and epigenome. Key technologies and findings used in investigations will be emphasized, along with highlights regarding the future frontiers of salivary molecular diagnostics. 

## 2. Properties of Saliva as a Diagnostic Fluid

Oral fluid, or saliva, is a clear, slightly acidic (pH = 6.0–7.0), and heterogeneous biological fluid consisting of secretions primarily from the parotid, submandibular, and sublingual glands [10]. The average daily flow of whole saliva is between 1 and 1.5 L. Physiologically, the function of saliva includes oral digestion, taste, lubrication, antibacterial protection, and buffering [10,11,12,13,14]. Saliva contains enzymes, hormones, antibodies, antimicrobial constituents, and cytokines—all of which are constituents gathered within the salivary glands and subsequently released into the oral cavity through small ducts by a cluster of cells called acini [11].

Many constituents are found to enter saliva from the blood as part of the endocrine system by transcellular (e.g., passive and active transport) or paracellular (e.g., extracellular ultrafiltration) means [15,16]. Because each of the salivary glands is encapsulated by capillaries, each gland is allowed free exchange of blood-based molecules into the adjacent saliva-producing acinus cells. Previous research has suggested that circulating biomolecules that originate from a diseased process from the bloodstream may eventually be transported into the salivary glands, which will then consequently modify and change the composition of saliva [3,4,5]. Hence, it is of recent interest to locate saliva-based targets to evaluate an individual’s current state of health, as saliva is functionally equivalent to blood plasma or serum in diagnostic medicine.

## 3. Biomarker Development and Clinical Reality

### Biomarker Background

The key to salivary diagnostics is the proper identification, validation, and detection of biomarkers related to disease. A biomarker refers to a quantifiable biological parameter that is measured and evaluated as an indicator of normal biological, pathogenic, or pharmacologic responses to a therapeutic intervention, according to the National Institutes of Health (NIH) [17]. This interaction may be functional or physiological, biochemical at a cellular level, or of molecular and conformational nature [18]. Biomarkers encompass a variety of classes: DNA, RNA, metabolites, proteins, and microbes. The usage of saliva as the diagnostic medium for molecular diagnostics of disease involves a thorough assessment of individual biomarkers and their relationship to one or more particular diseases, and also the study of an aggregate group of biomarkers to determine whether they can be used simultaneously for effective disease detection. This collection of biomarkers used for the diagnosis of disease is called a *molecular signature*. To properly identify the key markers that are correlated with a disease, researchers must perform a rigorous process of recruiting the appropriate patients, collecting samples in a uniform fashion, and performing discovery assays on specimens. There are specific considerations that must be made in each step of identifying and validating a salivary biomarker.

*Study Design:* The identification of a salivary biomarker begins with identifying a clinical scenario where saliva may be useful and then ensuring that the design of the study is rigorous enough for clinical practice. The prospective randomized open blinded end-point (PROBE) study design [19,20] is a developed framework that provides useful guidelines for conducting a study in a fashion that reduces bias and improves the likelihood of a positive outcome for biomarker development. The PROBE study design, for example, recommends that collection of samples be as close to the actual clinical scenario as possible for differentiation between a healthy and unhealthy subject using a biomarker. Further, as with any clinical study, proper weighing of the statistical power necessary for reaching validation is dependent on a host of factors, such as the feasibility of acquiring samples and the desired confidence levels that are hoped to be achieved by the study. It is critical that scientists and clinicians engage in active discussion to ensure that the clinical context for detection is well understood.

1. *Sample Collection and Processing:* Following the selection of the clinical scenario, the sample collection and processing phase is the next important phase of the study that must be well-regulated. In traditional collection of blood samples for testing of biomarkers, trained personnel must perform venipuncture, collect blood samples in vacuum tubes, and then process the samples to remove red blood cells. Saliva collection, inasmuch as it does not require venipuncture, can be done more conveniently and efficiently by laypeople and physicians, increasing the probability of a study’s success. However, in a similar fashion to blood-based testing, saliva-based testing still requires some specific parameters to be set by the study designer and to be uniformly applied during the collection phase of experimental work. Table 1 includes a preliminary assessment of some clinical considerations that must be made when collection saliva.
2. *Laboratory Analysis of Biomarkers*: Regardless of the specific molecular constituents that are being targeted (i.e., the test could be in relation to the proteome, transcriptome, genome, microbiome, metabolome, epigenome, etc.), the portion of study following study design and sample collection must take into account a proper laboratory workflow for processing the samples and ensuring that their sample quality is adequate for performing discovery or validation work. In this phase, it is necessary for the study to have well-designed quality control steps, thorough sample inventorying and storing (for future reference), and thorough documentation of the workflow for future reference and reproducibility. These steps must be taken in addition to the parameters that must be optimized for the specific technical procedures themselves.

## 4. Varieties of Biomarkers for Diagnostics in Saliva

There are many technologies employed to measure these biomarker targets, even within specific categories. The method for evaluation of proteomic, metabolomics, and microbiomic constituents include liquid chromatography, gel and capillary electrophoresis, nuclear magnetic resonance, immunoassays, magnetic bead immunoprecipitation, and mass spectrometry [24,25,26]. Each individual technique that is used has its respective merits and disadvantages for the various phases of a biomarker study. For example, in identifying genomic disease targets, techniques such as microarray or deep sequencing may be appropriate, but following the discovery of a biomarker scientists may want to change to a different technique such as quantitative polymerase chain reaction (qPCR) or droplet digital PCR (ddPCR), inasmuch as these may more suitable for the scales necessary for a definitive validation of a biomarker.

Biomedical research is a dynamic environment, with constant new developments and paradigms. There are always new frontiers opened up for investigation, partially because new technologies allow us to perform more detailed and sensitive detection of biomolecule targets, partially because our understanding of the biology of disease increases daily. Consequently, the specific molecular constituents that can be investigated for saliva diagnostics is an ever-increasing list. Nevertheless, at present it is possible to divide investigations of salivary molecular diagnostics into a few broad categories: proteomic, microbiome, immunologic, genomic (transcriptomic and epigenome), and metabolomics.

### 4.1. Proteomics

The proteome refers to all the individual proteins that may make up a biological system. A systemic study of all salivary secretory proteome (components with all the numerous post-translational modifications and protein–enzyme complexes) was implemented when the National Institute of Dental and Craniofacial Research (NIDCR) began to work towards a comprehensive catalogue of the human salivary proteome. In 2007, a collection of 1166 diverse proteins was recorded within the human salivary proteome [24,26]. This study was conducted using protein mass spectroscopy and 2D gel-electrophoresis, in addition to comparing these results with previously gathered protein databases.

While investigating the differences in salivary protein composition with that in plasma, Schulz et al. in 2013 reviewed a data set of proteins in plasma compiled by previous studies in the international Human Plasma Proteome Project, and found that approximately 30% of whole saliva proteins are found in plasma [27]. This overlap shows reasonable connections that can be drawn between the salivary proteome and different parts of the body [2]. As other studies have further shown, the salivary proteome can be used to draw connections between saliva and cystic fibrosis, diabetes, periodontitis, dental caries, and acquired immune deficiency syndrome (AIDS) [28,29,30,31,32]. Salivary proteomic analysis may even be useful for health safety applications such as radiation exposure [33,34,35]. A particularly compelling application of salivary proteomic biomarkers is the application to oral squamous cell carcinoma (OSCC). OSCC constitutes 90% of all cases of head and neck cancer. Despite the oral cavity being very accessible, most cases of OSCCs are not detected until the cancer has developed into advanced stages. There is much research effort dedicated to investigating salivary biomarkers as reliable early stage diagnostic analytes. As Cheng et al. and Yakob et al. have reported in their reviews of salivary biomarkers for oral cancer detection in 2014, more than 100 different salivary constituents have been suggested as potential OSCC markers [36,37]. Identified discriminatory cancer markers include matrix metalloproteinases (i.e., MMP1, MMP3, MMP9), cytokines (i.e., interleukin-6, interleukin-8, vascular endothelial growth factor A (VEGF-A), tumor necrosis factor α (TNF-α), transferrins, and fibroblast growth factors. Recently, Gleber-Netto et al. elucidated on and further confirmed results of previous studies, using ELISA and a two-marker univariate fractional polynomial (FP) model [38], and demonstrated that combining multiple salivary analytes allowed for the greatest degree of distinguishing oral squamous cell carcinoma from controls and also from other potentially malignant oral disorders. The combinatory models of IL-8 protein with IL1-β protein and of IL-8 protein with H3 histone family member 3A (*H3F3A*) mRNA both yielded an area under the curve (AUC) value of approximately 0.87. Additionally, mass spectrometry-based techniques have revealed that a combination of the protein markers—myeloid-related protein 14 (MRP14), profilin, cluster of differentiation 59 (CD59), catalase, and Mac-2-binding protein (M2BP)—yields a sensitivity of 90% and specificity of 83% for OSCC detection [39].

Saliva analysis has also been shown to diagnose more distal systemic malignancies such as breast cancer, lung cancer, ovarian cancer, and pancreatic cancer [40,41,42,43,44,45]. The main goal of these studies has been the discovery, verification, and validation of a panel of protein biomarkers so that they can be used in early detection of indicated diseases. Bigler et al. suggests that the protein expression of a receptor tyrosine kinase oncogene c-erbB-2 in saliva can be helpful to measure patient response to chemotherapy for treatment of breast cancer; Zhang et al. reports the de novo discovery and validation of eight mRNA biomarkers and one protein biomarker (carbonic anhydrase 6 (CA6) protein) for the noninvasive detection of breast cancer, drawing from two-dimensional difference gel electrophoresis, RT-qPCR, and protein immunoblot techniques [40,41]. Likewise, Xiao et al. utilized 2-D difference gel electrophoresis and mass spectroscopy to identify 16 candidate protein biomarkers to discriminate lung cancer patients from healthy control patients, and 3 of these (haptoglobin hp2 (HP), α2-glycoprotein (AZGP1), and human calprotectin) showed reliable discriminatory power, with 88.5% sensitivity and 92.3% specificity with an AUC of 0.90 [46]. 

At present, one of the major diseases being moved forward to definitive validation is primary Sjögren’s syndrome (SS). Patients with SS have a systemic autoimmune disease and suffer from irreversible damage of salivary and lacrimal glands. This damage is the result of progressive inflammation of the exocrine glands, stemming from an overexpression of human leukocyte antigen–antigen D related (HLA-DR) and lymphocytic infiltration of glandular tissue. This disease may occur as primary SS (pSS) or as a secondary disease associated with other autoimmune diseases such as rheumatoid arthritis (RA) or systemic lupus erythematosus (SLE). Researchers who study the proteomics of Sjögren’s syndrome look closely at the production of cytokines (i.e., interferons and interleukins) and the downstream signaling pathway molecules as overexpressed or underexpressed constituents of saliva, in afflicted individuals versus a control population. Using mass spectrometry, Hu et al. and other studies identified and further verified candidate proteins cathepsin D (CPD), α-enolase, and β2-microglobulin (B2M) as biomarkers that can discriminate primary SS from both systemic lupus erythematosus and healthy patients [43,44,45]. Additionally, statistical analysis showed that combining all three biomarkers yielded even higher sensitivity and specificity values compared to using them individually. At present, researchers at the UCLA School of Dentistry are engaged in a definitive validation of a panel of these SS biomarkers, evaluating a panel of markers with subjects at multiple clinical sites. A salivary biomarker test for Sjögren’s syndrome would be a valuable accessory to clinicians, as it would provide a noninvasive and convenient method of assessing SS without the need for biopsy. 

### 4.2. Immunomics

Using immunological markers of systemic infections to diagnose and prognosticate patients is another sector of investigation in salivary diagnostics. Immunomic approaches to the characterization and clinical identification of salivary biomarkers have been utilized by researchers for instances of infections from HIV virus, hepatitis A, B, and C. The aforementioned mechanisms of molecular transport of substances from blood serum into salivary gland ducts stem from the fact that salivary glands are highly vascularized and can uptake blood-based constituents. As such, the number of immunological markers (which are absorbed and subsequently secreted by the salivary glands into the oral cavity) are surprisingly comparable in concentration to immunological markers in the vascular system [44]. 

One development and application of salivary diagnostics in immunological salivary diagnostics is the marketing of FDA-approved testing kits for AIDS. Studies have also been conducted to determine whether or not the hepatitis A virus can be monitored through IgM, IgA, and IgG tests, and hepatitis B virus and hepatitis C virus through detection of altered IgG levels. Hepatitis A, B, and C are usually diagnosed through blood draws, but the noninvasive nature of saliva draws has its appeals. Although the immunoassays performed for hepatitis C virus antibodies possess an accuracy of 97.5%, the FDA has not approved of this technique yet. Finally, IgG antibodies directed against specific *Plasmodium falciparum* antigens, dengue virus antigens, and Ebola virus antigens can be also detected in saliva. Utilizing saliva to detect these disorders is a process that is well underway, but continued research is necessary to expand the now commercially available HIV tests to include a wider variety of microbes. 

In the research conducted at UCLA regarding the definitive validation of SS markers, research on SS in the proteome has been paired with immunologic analysis, as the two profiles present strong candidates for building a molecular profile for SS. Studies from many groups have demonstrated the presence of many autoantibodies in whole saliva collected from pSS patients, including IgA and IgM rheumatoid factor, anti-Ro/SSA, anti-La/SSB, anti-spectrin, anti-Ro52, anti-Ro60, anti-transglutaminase, and muscarinic acetylcholine receptor (mAChR) IgA [45,47,48,49,50,51]. These studies report that these pSS-related autoantibodies are able to discriminate patients with SS from both patients with systemic lupus erythematosus and healthy individuals. The use of SS immunological biomarkers to help diagnose, classify, and predict the prognosis of patients with pSS has never looked better, and may become an extremely helpful complement to investigations using other SS protein biomarkers. 

### 4.3. The Salivary Microbiome

Following the Human Microbiome Project, established by the NIH, many studies have investigated the amount of microbiological flora in the oral cavity, some estimating approximately 500–1000 bacterial species and others reporting up to 10,000 species in the mouth [3,52,53,54,55]. Common techniques employed by researchers are bacterial microarrays, DNA hybridization, PCR, next-generation sequencing, and quantitative 16S rRNA gene sequencing. Most recently and most promising is the employment of an oligonucleotide microarray based on 16S rRNA, aptly named human microbe identification microarrays (HOMIM) [3,52,53,54,55]. HOMIM has been used in studies using cohorts of individuals with disease versus control cohorts, in order to detect species of microbiota. The aim is to identify pathogenic profiles, monitor changes, and map the discovered alterations to diseases, of which many have been found to be correlated to microbiota. Consequently, such epidemiologic investigations can allow researchers to assess risk of disease based on HOMIM-based targeted characterization by comparing to already databased phyla or genera. Infectious diseases, local diseases, and systemic diseases that have been identified are HIV-1, HIV-2, hepatitis A, hepatitis B, hepatitis C, malaria, dengue fever, tuberculosis, Ebola virus disease, herpes simplex, disease caused by and correlated to Epstein-Barr virus, herpesviruses infection, cytomegalovirus-caused illnesses, oral cancer, Crohn’s disease, pancreatic cancer and chronic pancreatitis, periodontal disease, dental caries, and obesity [53,54,55,56,57,58,59,60,61,62,63,64,65,66,67,68,69,70,71,72].

Utilizing HOMIM, Farrell et al. evaluated the oral microbiota of individuals diagnosed with either pancreatic cancer or chronic pancreatitis and determined these microbiota as potential markers [72]. Following this discovery phase, verification was performed using qPCR on a broader range of subjects. This work showed the validation of two oral bacterial candidates, *Neisseria elongate* and *Streptococcus mitis*, as microbial biomarkers with high accuracy (96.4% sensitivity and 82.1% specificity) in distinguishing pancreatic cancer patients from healthy ones. 

As studies indicate, understanding how alterations in the microbiome relate to local and systemic diseases could allow for early detection and for proper prognosis and treatment for particular pathologies. Research conducted on the salivary microbiome is suggestive about the potential of correlating bacterial profiles in the saliva with different oral and systemic diseases.

### 4.4. Genomics—Transcriptomics and Epigenomics

Salivary transcriptomics is based on the analysis of the oral transcriptome—the set of all mRNA molecules that can be found in the salivary milieu. Broadly speaking, in order to analyze the transcriptome of a patient, saliva is collected and then DNA/RNA extraction is performed. Following the extraction, the DNA/RNA is amplified for analysis. After quantification of the nucleic acid content present in the saliva, comparisons can be made with known transcriptomic information. Technologies used to detect and analyze genomic and transcriptomic include gene chip arrays, DNA hybridization, qPCR, and gel electrophoresis [73].

An example study where the transcriptomic profile was examined in a patient population was conducted by Li et al. on a cohort of OSCC patients, extracting RNA from unstimulated cell-free saliva and then performing microarray analysis in order to identify candidate genes with altered expression levels [74]. Following the microarray study, seven mRNA markers that had higher expression levels were selected and tested for in remaining saliva samples using qPCR. This study found that a combination of these mRNA markers was able to yield a clinical sensitivity of 91% and a clinical specificity of 91%. For salivary biomarkers based on transcriptomics, breast cancer and Sjögren’s syndrome have also been identified and appropriately validated [41,75].

Salivary transcriptomic profiling has also been applied on a mouse model in order to better understand the salivary transcriptome. In a study by Gao et al., researchers compared the transcriptomes of the tumor, serum, salivary glands, and saliva on tumor-bearing mouse models and made comparisons with healthy control mice [76]. In this study, mouse saliva, blood, salivary glands, and tumor tissue were collected and RNA extraction was performed. The extracted DNA was then subject to microarray analysis to compare the transcriptomes of the samples with each other. These researchers found that the salivary transcriptome in diseased mouse samples had a high number of overlaps with the salivary gland transcriptomes. Their conclusion was that there are multiple sources of salivary mRNA. An understanding of these sources better allows researchers to target high concentrations of biomarkers and identify/validate the diseases associated with them.

An additional branch of investigation that has yielded fruitful results is the presence of microRNA in saliva. MicroRNA (miRNA) can be defined as a short RNA sequence, approximately 19–25 base pairs in length, that plays a role in the regulatory process of the cell by inhibiting transcription of sequences (consequently preventing protein synthesis) and causing messenger RNA degradation [77]. Clinical examination of miRNA has noted that miRNA sequences miR-125a and miR-200A are expressed lower in patients with oral squamous cell carcinoma [78]. Analysis of RNA in saliva using RNA sequencing has also led to the discovery of circular miRNA sequences [79].

In terms of oral epigenomics, the basis of the study is evaluating the changes and differences of downstream phenotypes or gene expression that are not a result of genetic heredity. Based on epigenetic differences in DNA regulation and histone modification, changes between a control subject and a diseased subject are easily discernible. In addition to epigenetic changes caused by the DNA regulation or histone modification, epigenetic changes may be a result of invading pathogens as well. Similar to transcriptomic analysis, qPCR of these epigenetic samples will yield differences between tumor and control samples [6]. As a result, by combining the analytical tools of transcriptomic analysis and the changes in oral epigenomics, a patient’s condition can be appropriately assessed. In an overarching view, both epigenomics and transcriptomic analysis all pertain to the basic genomic analysis of salivary biomarkers. By analyzing these biomarkers in the methods listed above for transcriptomic analysis, the genomic differences in patients can be identified and validated. 

### 4.5. Metabolomics

Metabolites are those small compounds that are the small-molecule products that exist as the body undergoes its metabolic processes [80]. Metabolomic investigation in saliva seeks to catalog these different small molecules in oral fluid and determine if they can be diagnostically useful for detection. To analyze the patient’s metabolic profile, multiple analytical platforms can be used to discern discrepancies in the patients’ saliva chemical fingerprint. These platforms include nuclear magnetic resonance spectroscopy, gas chromatography mass spectrometry, direct flow injection/liquid chromatography mass spectrometry, inductively coupled plasma mass spectrometry, and high-performance liquid chromatography [81]. Using a combination of diagnostic tools, Dame et al. was able to identify and annotate 308 salivary metabolites in human saliva [82]. A notable highlight of metabolomic research was conducted at the University of California Los Angeles by Sugimoto et al. in examining the metabolic profile of patients with lung cancer, breast cancer, pancreatic cancer, and diabetes [83]. These researchers utilized capillary electrophoresis time of flight mass spectroscopy, a technique which allowed the separation of different metabolites from saliva and enabled their profiling through the consequent application of mass spectroscopy. After analyzing the compound migration and mass spectroscopy data of the clinical specimens and fitting them to known mass-spec and mobility data using computational algorithms, 57 metabolites were selected as candidates that could be used for disease identifications. Some of the results of this study were quite encouraging, including pancreatic cancer having an AUC value of 0.993.

There are a number of additional applications for metabolomics analysis of saliva in diagnostics. Aimetti et al. [84] reported on periodontal disease metabolites in saliva, and Kageyama et al. [85] and Mikkonen et al. [86] have both conducted an examination of Sjögren’s syndrome metabolomics. Kageyama et al. [85] noted that Sjögren’s syndrome patients express a smaller set of metabolites compared to healthy controls [85], and Mikkonen et al. [86] performed analysis of nuclear magnetic resonance (NMR) spectroscopy, noting significantly increased levels of alanine and glycine. Furthermore, metabolomics monitoring has also been examined as a metric method for evaluating radiation exposure [87]. The results presented by these authors on salivary samples are suggestive works of the potential of salivary metabolomics profiling for a variety of disease and healthcare applications.

## 5. Electric Field-Induced Release and Measurement (EFIRM)

One of the most critical emerging technologies that has been explored for both mechanistic and clinical studies of salivary diagnostics is the electric field-induced release and measurement (EFIRM) method. This method is an electrochemical-based technique that emerged at the University of California, Los Angeles, out of research in developing biomarker detection tools for salivary diagnostics. 

There are a few distinct features that characterize the EFIRM methodology. Firstly, the platform is able to immobilize a high density of probes specific to circulating tumor DNA targets on a surface. These DNA probes are even able to specifically differentiate single base-pair mutations. The second predominant feature of the EFIRM is the usage of low-voltage electric fields to guide the hybridization process and lysing exosomes that contain key content. The third major feature of the method is the usage of an amplification step in order to specifically amplify the signal of the captured biomarker target and allow quantification through electrochemical reaction of the reaction between a peroxidase enzyme and tetramethylbenzidine (TMB) and hydrogen peroxide.

These features of EFIRM have allowed for it to be rapidly configured to a variety of specific contexts:

1. Basic Science: Complementing the diagnostic evaluation of saliva in a clinical setting is the need for rigorous scientific understanding of saliva’s relation to distal diseases. Specifically, this involves examining model systems (whether cell-based or animal-model based) in a rigorous and systematic fashion, which allows us to thoroughly understand the nature of salivary biomarkers and why biomarkers can often be found in the oral cavity. At present, there is intense interest in evaluating exosomes—microvesicular structures 30–100 nm in diameter found in saliva and other biofluids. They have been found to contain proteins, DNA, mRNA, and noncoding RNAs. Thus, some hypothesize that these exosomes may be the pathway where information is being carried from one portion of the body to another. Already, exosomes have been examined as prognostic markers for diseases such as lung cancer, squamous cell carcinoma, and breast cancer [42,88,89,90,91]. In examining exosomal entities as a possible transmitter of biomarkers to the oral cavity, EFIRM was used in conjunction with magnetic beads to extract exosomes from saliva, rapidly use electric fields to cause cargo unloading, and capture exosomal reference markers [88]. This method was used by Lau et al. for examining tumor-derived exosomes in a pancreatic cancer mouse model [42].
2. Translational Research: In regards to the clinical utility of the EFIRM method, EFIRM has been deployed on a number of clinical contexts. EFIRM was first deployed for successfully performing multiplexed targeting of the IL-8 protein and IL-8 mRNA markers for oral cancer [92]. More recently, EFIRM has taken an exciting direction forward by being able to detect nonsquamous cell lung cancer (NSCLC) oncogenic mutations, which determine the susceptibility of NSCLC to treatment by tyrosine kinase inhibitors. This examination of the ability to detect oncogenic mutations also showed high correlation. Most notably, EFIRM was able to successfully identify mutations in the endothelial growth factor receptor (EGFR) within saliva samples with a clinical sensitivity and specificity above 95% in two blinded cohort groups [93,94].

These results are indicative of the great potential of the EFIRM platform for performing detection of a variety of biomarker targets in different contexts. That this technique possesses the ability to perform noninvasive diagnostics with such extremely high sensitivity and specificity is an exciting development in the field of saliva diagnostics, and it demonstrates that saliva is quickly becoming a noninvasive, well-credentialed diagnostic medium that cannot be ignored. As the field of liquid biopsy is rapidly becoming more ascendant in the recent years, EFIRM has high potential as a liquid biopsy platform that can perform PCR-free rapid biodetection of oncogenic targets with a small volume of saliva, an exciting rapid point-of-care method amidst a field dominated by traditional technologies such as next-generation sequencing or PCR-based technologies [95].

## 6. Conclusions and Future Direction

This article has given a broad overview of the nature of research in molecular diagnostics in regard to saliva. The key fields of investigation for salivary diagnostics have been discussed and important studies have been highlighted. Studies already conducted show the great promise of different salivary biomarkers, and doubtless our ever-expanding knowledge of salivary biomarkers coupled with the potential present in combining multiple markers from different “omics” fields can possibly make molecular profiling for disease in saliva even more effective.

Also highlighted were some key trends to watch for in the field of salivary diagnostics. Studies that are working towards definitive validation of salivary biomarkers in large patient cohorts offer promise of rigorous validation that can approach the levels necessary for approval by the FDA. Furthermore, the usage of novel technologies such as the EFIRM platform shows the potential for rapid noninvasive detection of oncogenic mutations, demonstrating that saliva as a diagnostic medium is more than an academic interest: saliva is quickly becoming a biofluid that is well-credentialed, and convenient and noninvasive in its collection.

## Figures and Tables

**Table 1:** Considerations regarding the collection and sample processing steps of saliva.

Parameter	Description
Subject Status	Prior to collection of samples, study researchers should prescribe either fasted or unfasted states to patient cohorts. It has been observed that saliva in a fasted state may lead to differences in composition of saliva [21].
Sample Collection Time	When instructing patients on sample collection, it is necessary to specify a window of time that the patient may be allowed to contribute their saliva to a sample collection instance. These windows are important precautions against sample degradation if the time is long, and also allow adequate time for saliva to be collected with biomarker content.
Sample Collection Volume Requirement	Typically, running biomarker identifications or bioassays on a salivary sample will require a specific volume that must be collected for running tests. If the subject has a pathology that severely limits the flow of saliva to the oral cavity, it may be necessary for the study to have modifications made to account for the reduced volume that may be achievable.
Sample Collection Method	A multitude of different saliva collection methods can be used for testing. Typical collection protocol used at facilities such as UCLA involves the usage of traditional falcon tubes on ice, but saliva collectors have also been explored for collection [22]. This method can be designated as “unstimulated” since it uses saliva that has naturally pooled in the mouth. This is differs from the class of “stimulated” collection, where samples of saliva are attained through methods such as absorbent pads or chewing on parafilm [23]. The methods used must be appropriately identified, as results of analysis may differ depending on the saliva collection method.
Sample Processing and Storage	Collections of saliva must be properly optimized based on desired targets to be tested for. The inclusion of constituents in the saliva such as epithelial cells may contribute background that may hinder assessments of whether molecular targets are truly in the saliva. For this reason, centrifugation may be considered for removing cells and creating cell-free saliva. Stabilizing agents may be necessary for preservation of samples, depending on the target.
